# Lipids, fatty acids and hydroxy-fatty acids of *Euphausia pacifica*

**DOI:** 10.1038/s41598-017-09637-9

**Published:** 2017-08-30

**Authors:** Hidetoshi Yamada, Yuya Yamazaki, Seiji Koike, Mayuka Hakozaki, Nozomi Nagahora, Sayaka Yuki, Akira Yano, Koichiro Tsurumi, Takuji Okumura

**Affiliations:** 1 Iwate Biotechnology Research Center, 22-174-4 Narita, Kitakami Iwate, 024-0003 Japan; 2 Life Science Materials Laboratory, ADEKA Corporation, 7-2-34 Higashiogu, Arakawaku Tokyo, 116-8553 Japan; 3 Hachinohe Gakuin University, 13-98 Mihono, Hachinohe Aomori, 031-8588 Japan; 4 National Research Institute of Aquaculture, Japan Fisheries Reseach and Education Agency, 422-1 Nakatsuhamaura, Minami-ise Mie, 516-0193 Japan

## Abstract

*Euphausia pacifica* is a good candidate for a resource of marine n-3 PUFA. However, few reports exist of the lipid and fatty acid composition of *E. pacifica*. To examine the potential of *E. pacifica* as a resource of marine n-3 PUFA, we analyzed *E. pacifica* oil. We extracted lipids from *E. pacifica* harvested from the Pacific Ocean near Sanriku, Japan. Lipid classes of *E. pacifica* oil were analyzed by TLC-FID and the fatty acid composition of the oil was analyzed by GC/MS. Free fatty acids and hydroxy-fatty acids were analyzed by LC/QTOFMS. The lipid content of *E. pacifica* ranged from 1.30% to 3.57%. The ratios of triacylglycerols, phosphatidylcholine, phosphatidylethanolamine and free fatty acids in *E. pacifica* lipids were 5.3–23.0%, 32.6–53.4%, 8.5–25.4% and 2.5–7.0%, respectively. The content of n-3 PUFA in *E. pacifica* lipids was 38.6–46.5%. We also showed that *E. pacifica* contains unusual fatty acids and derivatives: C16-PUFAs (9,12-hexadecadienoic acid, 6,9,12-hexadecatrienoic acid and 6,9,12,15-hexadecatetraenoic acid) and hydroxy-PUFAs (8-HETE and 10-HDoHE). *E. pacifica* is a good resource of marine n-3 PUFA. Moreover, *E. pacifica* can provide C16-PUFA and hydroxy-PUFAs.

## Introduction

The common term ‘krill’ refers to *Euphausiids*, a group of small crustaceans that comprises over 80 species. *Euphausiids* are widespread in oceans worldwide and are dominant organisms in zooplankton populations. *Euphausiids* are important links in the marine food chain because they are omnivorous, representing the first trophic level. *Euphausia pacifica* (Pacific krill) is the most common krill species in the North Pacific Ocean. *E. pacifica* is also found across the Bering Sea, through the southern part of the Sea of Okhotsk and the Sea of Japan, extending southwards to around 30 °N. The southern limit for *E. pacifica* is at the 9.5 °C isotherm at a depth of 200 m^1^. *E. pacifica* is one of the few commercially harvested *Euphausiids*; however, more than 30,000 tons are harvested every year in Japan alone.

Epidemiological and clinical studies have shown various health benefits from the consumption of marine n-3 polyunsaturated fatty acids (PUFAs), which include eicosapentaenoic acid (EPA) and docosahexaenoic acid (DHA)^2–4^. The effects of marine n-3 PUFAs on various risk factors of cardiovascular disease are in particularly well documented^4^. Reports on these health benefits have led to an increase in demand for products containing marine n-3 PUFAs. At present, the main source of marine n-3 PUFAs is fish oil. However, fish oil is a restricted resource and therefore other sources of marine n-3 PUFAs are required. In terms of n-3 marine PUFA content and biomass, krill oil is one of the most suitable resources of marine n-3 PUFAs^5–7^. Krill oil is not only an adequate substitute for fish oil, it also contains substances not found in fish oil, such as the phospholipid form of n-3 PUFA and the antioxidant astaxanthin. Because the phospholipid form of n-3 PUFA is incorporated into plasma faster than the triglyceride form, krill oil increases omega-3 index at a lower dose in humans^5, 6^. At the present, krill oil usually refers only to Antarctic krill (*Euphausia superba*) oil; however, it has been reported that Pacific krill oil contains a large amount of n-3 PUFA^8^ and is therefore a good candidate for a resource of marine n-3 PUFA.

Recently, we identified 5-, 8-, 9-, 12-, and 18-hydroxyeicosapentaenoic acid (HEPEs) as ligands of peroxisome proliferator-activated receptors (PPARs) in dried *E. pacifica* ^9^. Next, we examined whether HEPEs were naturally contained in *E. pacifica* or produced by the drying process, and found that fresh *E. pacifica* or frozen *E. pacifica* stored at temperatures lower than −30 °C contained only 8-HEPE. Thus, 8-HEPE is the only HEPE contained naturally in *E. pacifica*, and other HEPEs (5-, 9-, 12-, and 18-HEPE) are by-products of the drying process. We also found that 8-HEPE reduces plasma and hepatic triglycerides in high-fat diet-induced obese mice^10^. Saito *et al*. reported that C16-polyunsaturated fatty acids (C16:2, C16:3, C16:4) are contained in *E. pacifica* ^8^. 8-HEPE and C16-polyunsaturated fatty acid are rare lipids and have scarcely been reported in other marine animals. These could suggest that krill have a unique system of lipid metabolism. However, the lipid metabolism in krill remains obscure. In this study, to evaluate *E. pacifica* oil as a resource of marine n-3 PUFA and to determine the fatty acid and hydroxy-fatty acid compositions in *E. pacifica*, we analyzed *E. pacifica* lipids and thereby identified C16-polyunsaturated- and hydroxy-fatty acids.

## Results

### Lipid content and lipid classes of *E. pacifica*

We extracted lipid from *E. pacifica*, *E. superba*, *Balanus rostratus Hoek*, and *Marsupenaeus japonicus*, and analyzed lipid class composition. The lipid content and lipid class composition of each species are summarized in Table 1. The lipid content of *E. pacifica* ranged from 1.30% to 3.57%. The *E. pacifica* lipids contained mainly triacylglycerol (TG, 5.3–23.0%), diacylglycerol (DG, 5.2–15.2%), phosphatidylcholine (PC, 32.6–53.4%), phosphatidylethanolamine (PE, 8.5–25.4%) and free fatty acids (FA, 2.5–7.0%). The lipid content in *E. pacifica* increased from February to April, as the lipid content increased, the TG composition in *E. pacifica* lipid also rose. The lipid content was higher in *E. superba* than in *E. pacifica*; especially, the amount of TG was high in *E. superba*.

**Table 1:** Lipid content and lipid class composition. *1 The collection date of *E. superba* was not defined.

Species	Date of collection	Tissue	Lipid content on wet basis (wt%)	Lipid class composition (%)				
				TG	FA	DG	PE	PC
*E. pacifica*	2016 March 20	Whole	2.39	23.0	7.0	5.2	14.9	32.6
	2017 February 26	Whole	1.30	5.3	4.9	15.2	25.4	48.6
	2017 March 28	Whole	1.94	6.2	4.7	13.2	17.4	50.7
	2017 April 10	Whole	2.70	13.2	2.5	9.3	13.7	53.4
	2017 April 24	Whole	3.57	19.1	4.4	7.3	8.5	48.5
*E. superba*	*^1^–	Whole	6.93	45.2	2.8	2.4	1.6	43.8
*Balanus rostratus Hoek*	2016 March 17	Ovary	9.5	53.1	n.d.	10.0	4.8	16.1
	2016 April 25	Ovary	21.5	57.0	n.d.	5.9	6.1	20.3
*Marsupenaeus japonicus*	2015 April 24	Ovary	5.2	44.3	n.d.	3.6	3.4	48.6
		Liver	11.9	89.8	n.d.	1.3	1.1	7.6
		Muscle	0.8	4.6	n.d.	22.1	18.1	53.3
		Gill	1.2	3.7	n.d.	21.4	19.5	54.1

### Fatty acid composition in *E. pacifica* lipid

The fatty acid composition of lipid from *E. pacifica*, *E. superba*, *Balanus rostratus Hoek*, and *Marsupenaeus japonicus* is summarized in Table 2. Palmitic acid, EPA and DHA were found to be the major fatty acids, these three fatty acid together accounted for more than 60% of the fatty acid composition of *E. pacifica* lipid. From February 26 to April 24 in 2017, the relative composition of palmitic acid and palmitoleic acid increased, whereas that of DHA decreased. In *E. pacifica* lipid, the population of marine n-3 PUFA was more than 35%, which was higher than that in *E. superba* lipid, *Balanus rostratus Hoek* lipid, and *Marsupenaeus japonicus* lipid.

**Table 2:** Fatty acid composition in lipids.

Species	Date of collection	Tissue	Fatty acid composition in total lipids (%)								
			Palmitic acid (C16:0)	Palmitoleic acid (C16:1 (9))	Stearic acid (C18:0)	Oleic acid (C18:1 (9))	11-octadecenoic acid (C18:1 (11))	α-Linoleic acid (C18:3 (9, 12, 15))	Arachidonic acid (C20:4 (5, 8, 11, 14))	EPA (C20:5 (5, 8, 11, 14, 17))	DHA (C22:6 (4, 7, 10, 13, 16, 19))
*E. pacifica*	2016 March 20	Whole	18.2	2.4	1.7	9.9	7.1	1.8	2.4	20.9	22.2
	2017 February 26	Whole	21.1	1.6	2.5	10.7	7.1	1.7	2.1	21.2	25.3
	2017 March 28	Whole	21.2	3.0	2.1	7.1	6.9	1.6	2.1	27.4	18.3
	2017 April 10	Whole	22.8	5.9	2.0	7.0	8.2	n.d.	3.7	31.7	11.0
	2017 April 24	Whole	23.2	8.6	2.1	7.2	7.8	n.d.	3.3	29.2	9.4
*E. superba*	*^1^−	Whole	26.6	7.3	2.3	14.3	7.4	n.d.	n.d.	15.0	7.3
*Balanus rostratus Hoek*	2016 March 17	Ovary	18.6	8.5	16	4.5	5.3	n.d.	1.5	23.5	1.3
	2016 April 25	Ovary	13.8	19.1	2	5.1	13.8	n.d.	1.9	32.4	2.1
*Marsupenaeus japonicus*	2015 April 24	Ovary	20.6	8.8	6.2	13.8	5.5	n.d.	4.3	15.7	12.1
		Liver	21.8	3.7	5.8	1.9	14.5	n.d.	4.5	7.9	8.3
		Muscle	18.1	6.3	10.8	13.3	4.1	n.d.	5.8	16.3	13.1
		Gill	16.9	4.3	8.6	0.9	13.2	n.d.	7.0	16.3	11.7

### Free fatty acids in *E. pacifica*

**Figure 1:**
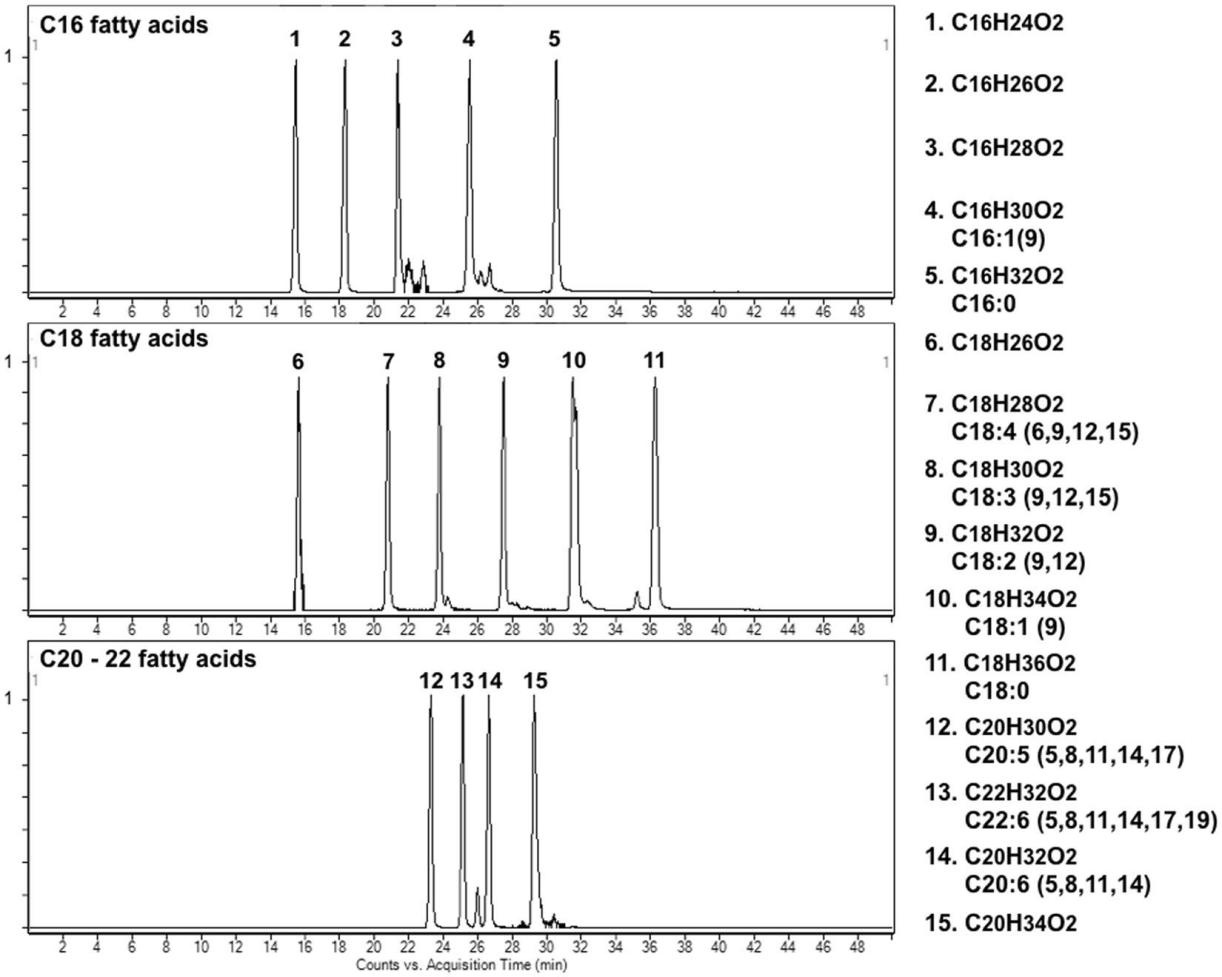
Extract chromatogram of free fatty acids contained in *E. pacifica*. Fatty acids in *E. pacifica* oil were analyzed by LC/QTOFMS. Fatty acids were predicted form the extracted ionized compounds by exact mass and molecular formula, and compounds were confirmed by using fatty acid standards. The compounds identified by using standards are shown with their molecular formula and structure. The compounds that were not identified by standards are shown with molecular formula only.

**Figure 2:**
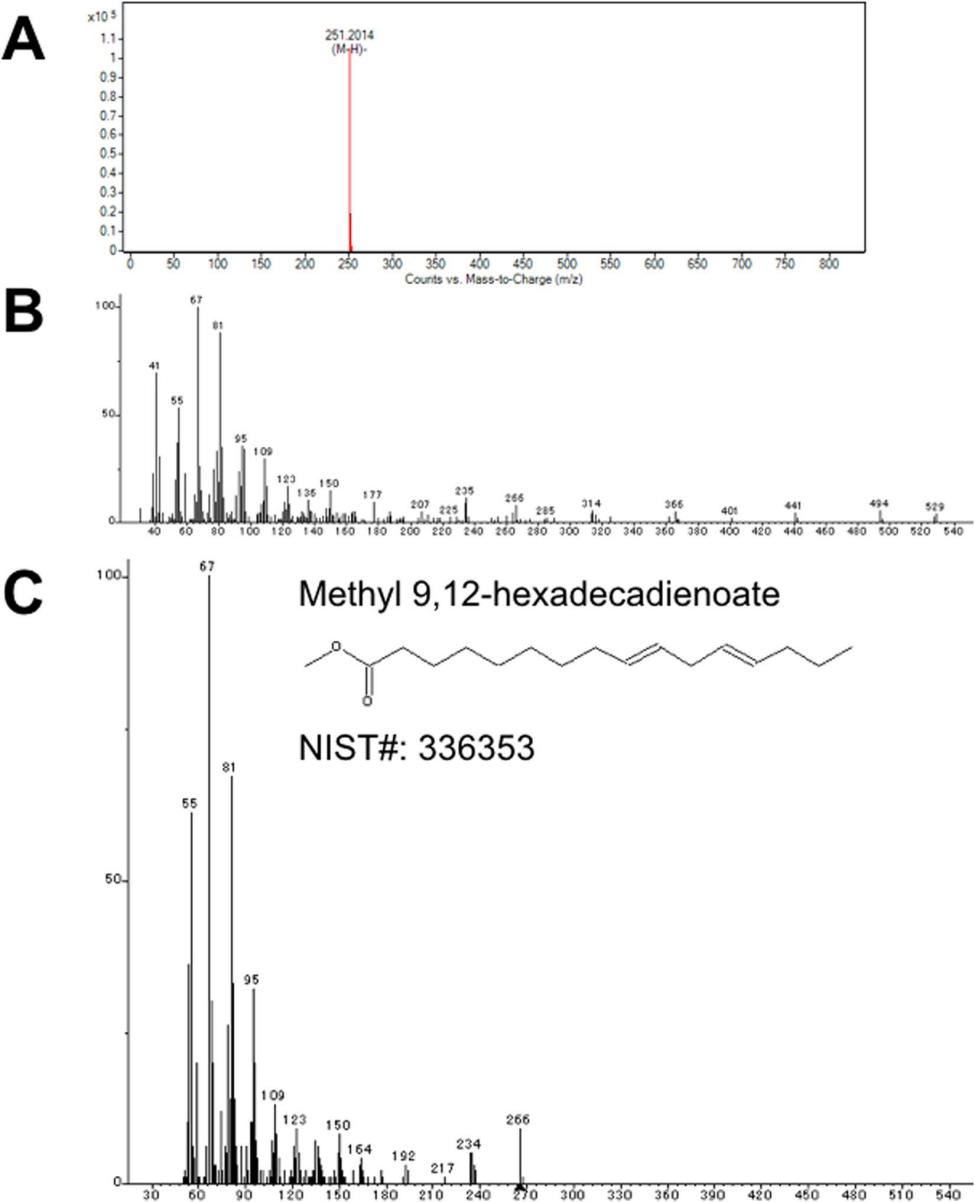
Structure analysis of 9,12-hexadecadienoic acid. We purified and analyzed the compound predicted as a C16H28O2 fatty acid. The exact mass and molecular formula of the compound were identified by using LC/QTOFMS. Fatty acid structure was identified by GC/MS. (**A**) Mass to charge (m/z) acquisition by LC/QTOFMS. (**B**) Mass spectra acquisition by GC/MS. (**C**) The result to access mass spectra for NIST2.0 library.

**Figure 3:**
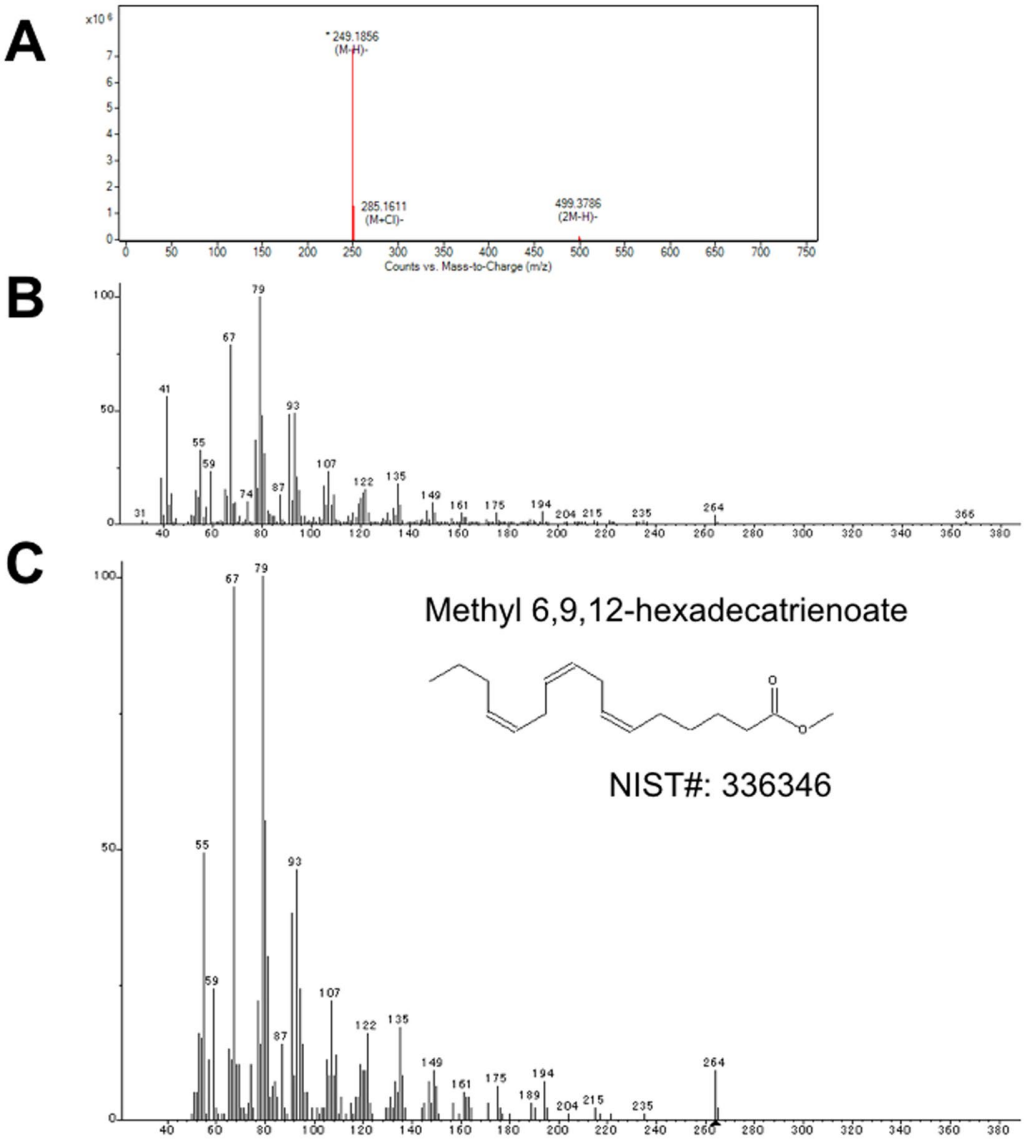
Structure analysis of 6,9,12-hexadecatrienoic acid. We purified and analyzed the compound predicted as a C16H26O2 fatty acid. (**A**) Mass to charge (m/z) acquisition by LC/QTOFMS. (**B**) Mass spectra acquisition by GC/MS. (**C**) The result to access mass spectra for NIST2.0 library.

**Figure 4:**
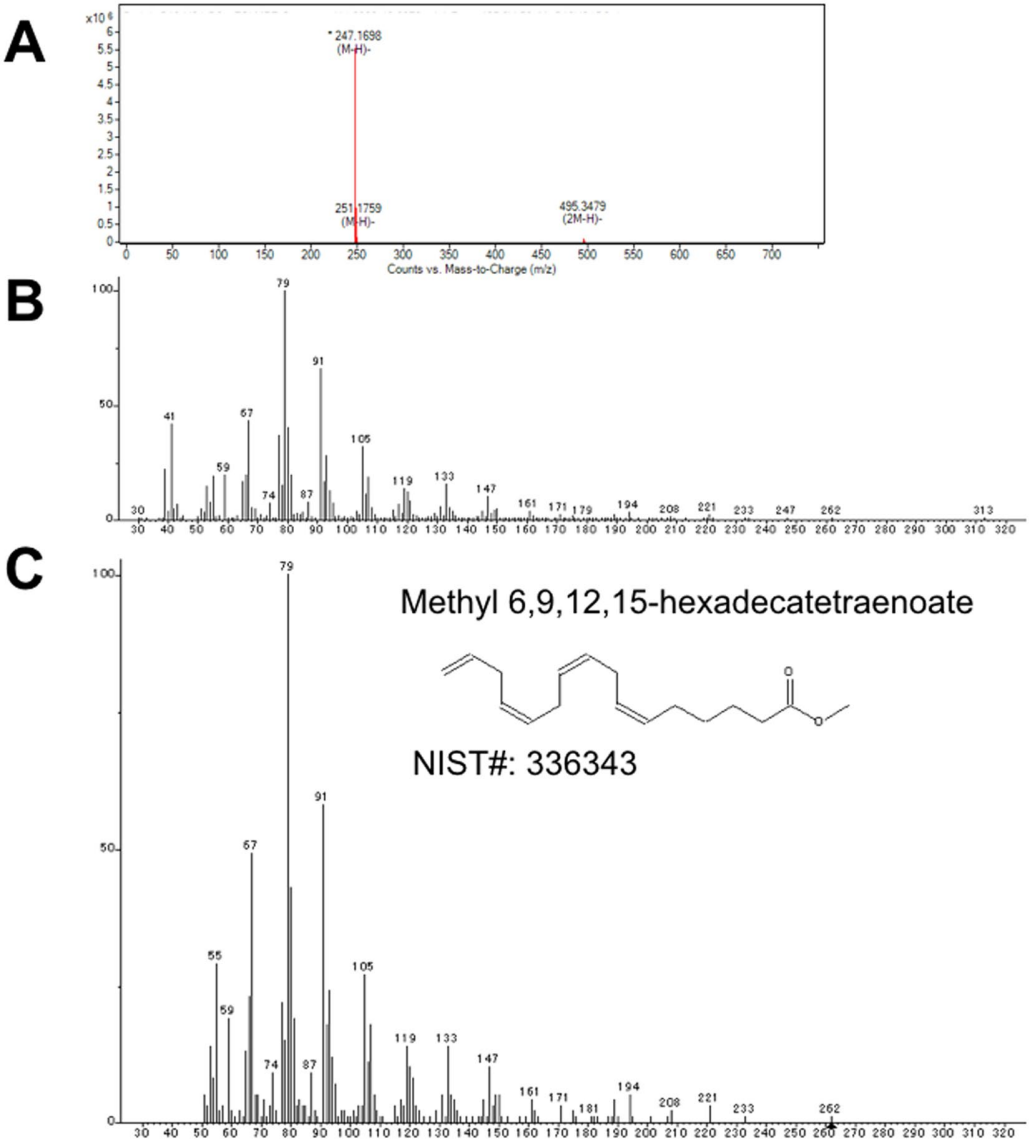
Structure analysis of 6,9,12,15-hexadecatetraenoic acid. We purified and analyzed the compound predicted as a C16H24O2 fatty acid. (**A**) Mass to charge (m/z) acquisition by LC/QTOFMS. (**B**) Mass spectra acquisition by GC/MS. (**C**) The result to access mass spectra for NIST2.0 library.

Saito *et al*. reported that *E. pacifica* oil contained five saturated fatty acids, nine monounsaturated fatty acids and thirteen polyunsaturated fatty acids. To examine the fatty acids contained in *E. pacifica*, we analyzed fatty acids by LC/QTOFMS. We detected four saturated fatty acids, two monounsaturated fatty acids and twelve polyunsaturated fatty acids as free fatty acids in *E. pacifica*. The carbon lengths of the fatty acids detected in *E. pacifica* were C12, C14, C16, C18, C20 and C22. In C12 and C14 fatty acids, only saturated fatty acid was detected. In C16 fatty acid, five types of fatty acid, each with different degrees of unsaturation, were detected (Fig. 1). Palmitic acid (C16:0) and palmitoleic acid (C16:1(9)) were identified using standards. To identify the C16:2, C16:3 and C16:4 fatty acids predicted by exact mass, we purified these fatty acids from *E. pacifica*. The methylated fatty acids were then analyzed by GC/MS. We searched for the detected ion fragment patterns in the NIST library and identified the C16:2, C16:3 and C16:4 fatty acids as 9,12-hexadecadienoic acid (C16:2 (6,9)) (Fig. 2), 6,9,12-hexadecatrienoic acid (C16:3 (6,9,12)) (Fig. 3) and 6,9,12,15-hexadecatetraenoic acid (C16:4 (6, 9, 12, 15)), respectively (Fig. 4). In C18 fatty acid, six types of fatty acid were detected. Stearic acid (C18:0), oleic acid (C18:1 (9)), linoleic acid (C18:2 (9,12)), alpha-linoleic acid (C18:3 (9, 12, 15)) and stearidonic acid (C18:4 (6, 9, 12, 15)) were identified using standards. The other fatty acid predicted to be C18:5 by exact mass was purified and analyzed using GC/MS. However, we did not find any compound with the same ion fragment pattern in the NIST library. The compound with the most similar ion fragment pattern in the NIST library was DHA. In C20 and C22 fatty acids, four types of fatty acid dihomo-gamma linoleic acid (C20:3 (8,11,14)), arachidonic acid (C20:4 (5,8,11,14)), EPA (C20:5 (5,8,11,14,17)), and DHA (C22:6 (4,7,10,13,16,19)) were detected.

The quantities of free fatty acids detected in *E. pacifica* are shown in Table 3. The major free fatty acids in *E. pacifica* were palmitic acid, oleic acid, EPA, and DHA. The content of palmitoleic acid, oleic acid, arachidonic acid and EPA in *E. pacifica* was high in samples collected on April 24 2017. In a comparison of *E. pacifica* with *E. superba*, the palmitoleic acid and arachidonic acid content was clearly higher in *E. pacifica*, whereas the palmitic acid was higher in *E. superba* than in *E. pacifica*. As compared with *Balanus rostratus Hoek* and *Marsupenaeus japonicus*, the EPA and DHA content was much higher in *E. pacifica*.

**Table 3:** Free fatty acid content. n.d. means not detected.

Species	Date of collection	Tissue	Content of free fatty acids on wet basis (µg/g)						
			Palmitic acid (C16:0)	Palmitoleic acid (C16:1 (9))	Oleic acid (C18:1 (9))	α-Linoleic acid (C18:3 (9, 12, 15))	Arachidonic acid (C20:4 (5, 8, 11, 14))	EPA (C20:5 (5, 8, 11, 14, 17)	DHA (C22:6 (4, 7, 10, 13, 16, 19)
*E. pacifica*	2016 March 20	Whole	389.9	25.2	168.5	23.0	36.5	559.0	422.6
	2017 February 26	Whole	510.1	12.6	256.2	5.7	55.9	632.0	602.6
	2017 March 28	Whole	385.9	45.7	384.8	18.6	77.5	1606.4	437.0
	2017 April 10	Whole	396.5	53.7	378.8	2.2	104.5	1409.8	239.4
	2017 April 24	Whole	559.6	125.0	605.0	2.0	215.7	2561.3	380.6
*E. superba*	*^1^–	Whole	1590.0	n.d.	430.5	8.2	n.d.	1075.4	342.0
*Balanus rostratus Hoek*	2016 March 17	Ovary	1.2	0.2	0.4	n.d.	n.d.	4.7	n.d.
	2016 April 25	Ovary	1.4	0.3	0.6	n.d.	0.1	17.5	0.3
*Marsupenaeus japonicus*	2015 April 24	Ovary	311.1	n.d.	n.d.	3.1	n.d.	40.3	n.d.
		Liver	770.2	n.d.	28.6	7.2	n.d.	52.0	n.d.
		Muscle	85.9	n.d.	4.8	0.5	n.d.	9.7	n.d.
		Gill	84.7	n.d.	26.4	0.8	0.7	30.6	21.1

### Hydroxy-fatty acids in *E. pacifica*

**Figure 5:**
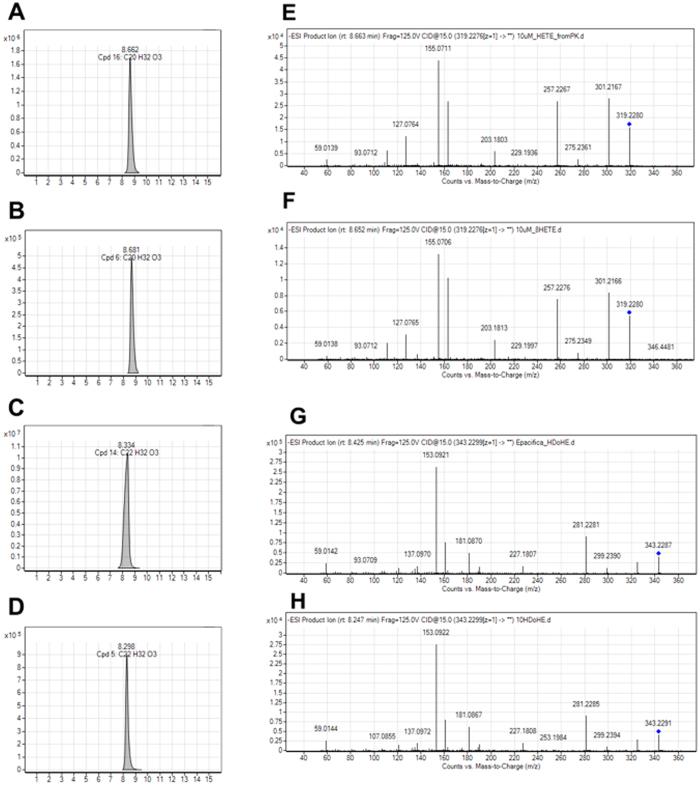
MS/MS analysis of 8-HETE and 10-HDoHE. (**A**–**D**) Chromatogram of LC/QTOFMS. (**E**–**H**) MS/MS spectra acquisition by LC/QTOFMS. (**A** and **E**) Compound C20H32O3 purified from *E. pacifica*. (**B** and **F**) 8-HETE standard. (**C** and **G**) Compound C22H32O3 purified from *E. pacifica*. (**D** and **H**) 10-HDoHE standard.

We identified 8-HEPE as a PPAR ligand from *E. pacifica* in a previous study. In this study, to examine whether *E. pacifica* contains other hydroxy-fatty acids, we analyzed *E. pacifica* extracts by LC/QTOFMS. We searched for hydroxy-fatty acids in *E. pacifica* extracts by exact mass and estimated molecular formulas and found the potential compounds HETE (m/z = 319.228, RT = 8.6) and HDoTE (m/z = 343.228, RT = 8.3) (Fig. 5A–D). To identity these compounds, we analyzed the MS/MS fragments and compared them with HETEs and HDoTE standards (Fig. 5E–H). As a result, we determined that both 8-HETE and 10-HDoHE are present in *E. pacifica*. The quantities of hydroxy-fatty acids and 8-HEPE in *E. pacifica* are summarized in Table 4. The content of 8-HEPE gradually increased from February to April. In *E. pacifica*, the 8-HEPE content was higher than that of 8-HETE and 10-HDoHE. Furthermore, the 8-HEPE content was much higher in *E. pacifica* than in *E. superba*, *Balanus rostratus Hoek* or *Marsupenaeus japonicus*

**Table 4:** Hydroxyl fatty acid contents. n.d. means not detected.

Species	Date of collection	Tissue	Content of hydroxyl fatty acids on wet basis (µg/g)		
			8-HEPE	8-HETE	10-HDoHE
*E. pacifica*	2017 February 26	Whole	14.6	2.4	4.0
	2017 March 28	Whole	11.8	0.6	1.1
	2017 April 10	Whole	18.5	1.1	1.2
	2017 April 24	Whole	29.2	2.4	1.9
*E. superba*	*^1^ -	Whole	0.86	n.d.	n.d.
*Balanus rostratus Hoek*	2016 March 17	Ovary	0.1	n.d.	n.d.
	2016 April 25	Ovary	0.2	n.d.	n.d.
*Marsupenaeus japonicus*	2015 April 24	Ovary	n.d.	n.d.	n.d.
		Liver	n.d.	n.d.	n.d.
		Muscle	n.d.	n.d.	n.d.
		Gill	n.d.	n.d.	n.d.

**Figure 6:**
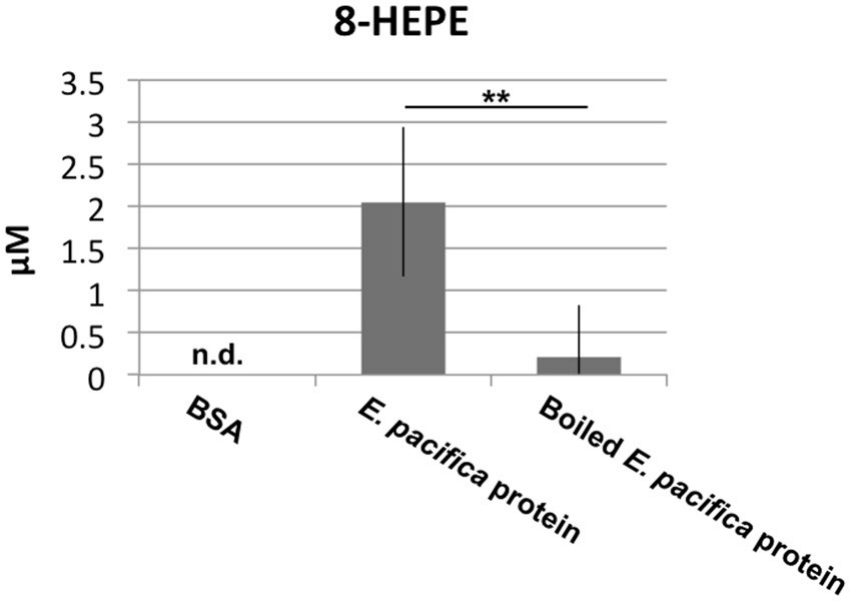
8-HEPE is produced from EPA by an enzymatic reaction. BSA, *E.pacifica* protein or boiled *E pacifica* protein (50 µg) was incubated with 2 nmol of EPA at 20 °C for 1 hour. The concentration of 8-HEPE produced was measured by LC/QTOFMS. Values are the mean and s.d. of five individual experiments. ** indicates p < 0.01.

8-HEPE, 8-HETE and 10-HDoHE share a common hydroxyl at the n-12 carbon position. To examine whether 8-HEPE is produced from EPA by enzymatic oxidation, we incubated fresh or boiled *E. pacifica* protein with EPA and determined the amount of 8-HEPE (Fig. 6). We found that 8-HEPE was produced by incubating *E. pacifica* protein with EPA, and the amount of 8-HEPE produced was significantly decreased by heating *E. pacifica* protein at 95 °C for 5 min.

## Discussion

In this study, we showed that the oil content of *E. pacifica* was 1.30–3.57%, approximately half of which consisted of phosphatidylcholine and phosphatidylethanolamine. Palmitic acid, EPA, and DHA were the major fatty acids that compromised the *E. pacifica* oil. The content of n-3 PUFAs was 38.6–46.5% (Table 2). In previous reports, the lipid content of *E. pacifica* caught near the Ogasawara Coast, Miyagi, and Hokkaido was determined as 0.59–1.75%, 3.34–6.41%, and 1.1–3.2%, respectively, on a wet weight basis^8, 11^. In each area, the lipid content of *E. pacifica* was high in spring (March – May) as compared with any other season. In spring, phospholipids accounted for more than 40% of the lipid content of *E pacifica*, and the content of n-3 PUFA was more than 29%. These results indicate that *E. pacifica* lipid composition has remained stable for at least the past 20 years, and that *E. pacifica* caught in spring is a good resource of the phospholipid form of n-3 PUFA.

It has been shown in a previous study that *E. pacifica* contains C16-PUFAs (C16:2 n-4, C16:3 n-4 and C16:4 n-1)^8^, however, chemical structure of these C16-PUFAs were not identified. In this study, we showed that the C16-PUFAs contained in *E. pacifica* were 9,12-hexadecadienoic acid, 6,9,12-hexadecatrienoic acid and 6,9,12,15-hexadecatetraenoic acid (Figs 2–4). C16-PUFAs are unusual fatty acids and are not present in *E. superba* or *Euphausia crystallorophias* ^12^. 9,12-Hexadecadienoic acid and 6,9,12-hexadecatrienoic have been reported in *Phaeodactylum ticornutum* ^13^ and *Acanthameba castellanii* ^14^. 6,9,12,15-Hexadecatetraenoic acid has been reported only in marine phytoplankton^12^ and *Acanthameba castellanii* ^14^. It is not certain whether the C16-PUFAs are synthesized in *E. pacifica* or derived from phytoplankton. This question will be answered when the *E. pacifica* genome is decoded.

We previously reported that *E. pacifica* contains 8-HEPE^9^. In this study, we demonstrated that *E. pacifica* also contains 8-HETE and 10-HDoTE (Fig. 5). Prior to our previous study^9^, hydroxy-PUFAs had not been reported in krill. However, prostanoids, oxylipins, cyclooxygenase and lipoxigenase had been reported in crustaceans, and some biological functions have been predicted^15–18^. Our present results indicate that *E. pacifica* has a lipoxygenase that can oxidize the n-12 carbon of EPA, AA, and DHA (Fig. 6). The 8-HEPE contents in *E. pacifica* was increased from February to April (Table 3); thus, it seems that 8-HEPE might contribute to *E. pacifica* growth and maturation. Identification of the *E. pacifica* lipoxygenase and biochemical analysis will clarify the biological function of hydroxy-PUFAs. In addition, the identification of 8-HEPE synthetic pathways in *E. pacifica* might provide a way to produce 8-HEPE artificially.

## Conclusions

To meet the continuing increasing demand for marine n-3 PUFA globally, *E.pacifica* might represent a new resource of the phospholipid form of n-3 PUFA. Moreover, *E. pacifica* can provide unusual fatty acids, C16-PUFA and hydroxy-PUFAs that cannot be obtained from other resources.

## Materials and Methods

### Materials

*E. pacifica* was purchased from Kawashu Inc. (Iwate, Japan) and Kokuyou Inc. (Iwate, Japan), and was originally harvested from the Pacific Ocean near Iwate, Japan. *E. superba* was purchased from HiromatsuKyu Fishery Co.,Ltd (Fukuoka, Japan). *Balanus rostratus Hoek* was purchased directly from a fisherman culture in Ofunato, Iwate, Japan. *Marsupenaeus japonicus* was purchased from a local fisherman in Aichi, Japan. Fatty acids, HEPEs, hydroxyeicosatetraenoic acids (HETEs) and hydroxy docasahexaenoic acids (HDoHEs) were purchased from Cayman Chemical (Ann Arbor, MI). Dipalmitoyl Phosphocholine, 1,2-dipalmitoyl-sn-glycero-3-phosphoethanolamine, glyceryl tripalmitate, DL-a-palmitin and 1-palmitoyl-sn-glycero-3-phosphocoline were purchased from Sigma-Aldrich (St. Louis, MO).

### Lipid extraction and the analysis of lipid classes

Lipids were extracted from *E. pacifica, E. superba, Balanus rostratus Hoek, and Marsupenaeus japonicus* according to the Bligh-Dyer method^19^. Lipid classes were analyzed by silica gel thin-layer chromatography (TLC). First, 5 µg of lipid extract was applied to a Chromarod (LSI Medience, Tokyo, Japan). Next, chloroform/methanol/water (42:24:2.5) was drawn 5 cm up the Chromarod. After drying the Chromarod, hexane/diethyl ether (50:30) was drawn 10 cm up the Chromarod. Lipids were detected and measured by TLC-FID (LSI Medience). Dipalmitoyl Phosphocholine, 1,2-dipalmitoyl-sn-glycero-3-phosphoethanolamine, glyceryl tripalmitate, DL-a-palmitin and 1-palmitoyl-sn-glycero-3-phosphocoline were used as lipid standards.

### Gas chromatography/mass spectrometry (GC/MS)

Triacylglycerol (TAG) and phospholipid (PL) were esterified by incubation with methanol containing 5% hydrochloric acid at 50 °C for 30 min. Analysis of the fatty acid methyl esters was performed on a gas chromatography (Agilent Technologies 6890 N)/mass spectrometry (Agilent Technologies 5975B) system using with a DB-23 gas chromatography column (60 m × 0.25 mm i.d. and 0.15-µm film thickness [Agilent technologies]). Helium (carrier gas) was passed through the column at a constant linear velocity of 40.0 mL/min, and the split ratio was 50. The initial oven temperature was maintained at 50 °C for 1 min, increased to 175 °C at a rate of 25 °C/min, increased to 230 °C at a rate of 4 °C/min, and then maintained for 5 min. The temperatures of the inlet, interface, and ion source were 250 °C, 280 °C and 230 °C, respectively. Electron impact (EI, 70 eV) was used as the ionization mode.

MS data were analyzed with Agilent GC/MSD ChemStation software. MS fragment data were searched for in NIST atomic spectra database 2.0.

### Liquid chromatography/hybrid quadrupole time of flight mass spectrometry (LC/QTOFMS)

HEPEs, HETEs and HDoTEs were separated on an InertSustain ODS-3 column (2.0 mm dia. × 250 mm; GL Science Inc.) with gradient elution (water containing 10 mM ammonium acetate/acetonitrile, 55/45 to 5/95 in 25 min) at a flow rate of 0.2 mL/min. The temperature of the column was kept at 40 °C. The compounds were identified and quantified by LC/QTOFMS (Agilent Technologies) using Agilent Mass Hunter Workstation Software (Agilent Technologies). The velocity of the drying gas was 10 L/min. The temperature of the gas was 325 °C. The voltages of Vcap, fragmentor, and skimmer were 3500, 125 and 65 V, respectively. The pressure of the nebulizer was 30 psig.

### *E. pacifica* protein extraction and enzyme reaction

*E. pacifica* was homogenized in a 3-fold higher volume of Tris-HCl buffer (pH 7.4) on ice and centrifuged at 20,000 g for 10 min at 4 °C. Next, *E. pacifica* protein in the liquid layer was concentrated by Amicon Ultra 10 K (Merck Millipore). The concentration of *E. pacifica* protein was measured by Coomassie Plus Reagent (Thermo Scientific), and then 50 µg of *E. pacifica* protein and 2 nmol EPA were incubated in 20 µL of Tris-HCl (pH7.4) buffer at 20 °C for 1 hour. After the enzymatic reaction, 60 µL of acetonitrile containing 1% of formic acid was added to the samples and mixed. Next, the samples were centrifuged at 20,000 g for 10 min at room temperature. The 8-HEPE concentration of the liquid layer was measured by LC/QTOFMS.
